# A cross-sectional study of determinants of indoor environmental exposures in households with and without chronic exposure to biomass fuel smoke

**DOI:** 10.1186/1476-069X-13-21

**Published:** 2014-03-24

**Authors:** Suzanne L Pollard, D’Ann L Williams, Patrick N Breysse, Patrick A Baron, Laura M Grajeda, Robert H Gilman, J Jaime Miranda, William Checkley

**Affiliations:** 1 Division of Pulmonary and Critical Care, School of Medicine, Johns Hopkins University, 1800 Orleans Ave, Suite 9121, Baltimore, MD, USA; 2 Program in Global Disease Epidemiology and Control, Department of International Health, Johns Hopkins University, Bloomberg School of Public Health, Baltimore, MD, USA; 3 Department of Environmental Health Sciences, Johns Hopkins University, Bloomberg School of Public Health, Baltimore, MD, USA; 4 CRONICAS Center of Excellence in Chronic Diseases, Universidad Peruana Cayetano Heredia, Lima, Peru; 5 Departamento de Medicina, Escuela de Medicina, Universidad Peruana Cayetano Heredia, Lima, Peru

**Keywords:** Biomass smoke, Biomass fuel, Cookstoves, Biomarkers, Exhaled carbon monoxide, Environmental exposure

## Abstract

**Background:**

Burning biomass fuels indoors for cooking is associated with high concentrations of particulate matter (PM) and carbon monoxide (CO). More efficient biomass-burning stoves and chimneys for ventilation have been proposed as solutions to reduce indoor pollution. We sought to quantify indoor PM and CO exposures in urban and rural households and determine factors associated with higher exposures. A secondary objective was to identify chronic vs. acute changes in cardiopulmonary biomarkers associated with exposure to biomass smoke.

**Methods:**

We conducted a census survey followed by a cross-sectional study of indoor environmental exposures and cardiopulmonary biomarkers in the main household cook in Puno, Peru. We measured 24-hour indoor PM and CO concentrations in 86 households. We also measured PM_2.5_ and PM_10_ concentrations gravimetrically for 24 hours in urban households and during cook times in rural households, and generated a calibration equation using PM_2.5_ measurements.

**Results:**

In a census of 4903 households, 93% vs. 16% of rural vs. urban households used an open-fire stove; 22% of rural households had a homemade chimney; and <3% of rural households participated in a national program encouraging installation of a chimney. Median 24-hour indoor PM_2.5_ and CO concentrations were 130 vs. 22 μg/m^3^ and 5.8 vs. 0.4 ppm (all p<0.001) in rural vs. urban households. Having a chimney did not significantly reduce median concentrations in 24-hour indoor PM_2.5_ (119 vs. 137 μg/m^3^; p=0.40) or CO (4.6 vs. 7.2 ppm; p=0.23) among rural households with and without chimneys. Having a chimney did not significantly reduce median cook-time PM_2.5_ (360 vs. 298 μg/m^3^, p=0.45) or cook-time CO concentrations (15.2 vs. 9.4 ppm, p=0.23). Having a thatched roof (p=0.007) and hours spent cooking (p=0.02) were associated with higher 24-hour average PM concentrations. Rural participants had higher median exhaled CO (10 vs. 6 ppm; p=0.01) and exhaled carboxyhemoglobin (1.6% vs. 1.0%; p=0.04) than urban participants.

**Conclusions:**

Indoor air concentrations associated with biomass smoke were six-fold greater in rural vs. urban households. Having a homemade chimney did not reduce environmental exposures significantly. Measures of exhaled CO provide useful cardiopulmonary biomarkers for chronic exposure to biomass smoke.

## Background

More than half the world population uses solid fuels indoors for cooking and home heating. Incomplete combustion of these materials results in the production of hazardous air pollutants that affect respiratory health [1,2]. The World Health Organization has identified indoor combustion of biomass solid fuels as the fourth leading risk factor for disease burden worldwide [3]. In 2010, indoor air pollution caused an estimated 4 million premature deaths and is the most important environmental risk factor globally and in poor regions of the world [4]. Biomass smoke exposure is highest among women and children, as these groups generally spend more time in the home and in areas designated for cooking.

Particulate matter (PM), especially particles <2.5 μm in diameter (PM_2.5_), is a key component of biomass fuel smoke. Exposure to PM from biomass combustion has important effects on the development of respiratory diseases. In particular, exposure to biomass smoke is associated with acute respiratory infections in children, lung cancer, chronic obstructive pulmonary disease, asthma, and cardiovascular disease, among others [5-17]. Of these, COPD is most highly correlated with air pollution exposure [18]. Carbon monoxide (CO) is also produced by the incomplete combustion of carbon-containing biomass fuels. CO results in tissue hypoxia due to its higher affinity to hemoglobin as compared to oxygen; thus, carboxyhemoglobin and oxygen saturation levels in the blood are key indicators in determining the effects of acute CO exposure. CO also acts through direct CO-mediated damage at the cellular level [19]. Acute exposure to high levels of CO can cause serious neuropsychiatric damage and can be lethal. In addition, chronic exposure to CO can have important health consequences, especially in those with underlying conditions such as anemia, asthma, and coronary artery disease, which impair tissue oxygenation [20]. Given the high burden of disease attributable to biomass fuel use, there is considerable interest in the design of interventions, such as chimney stoves, for reducing exposures to indoor biomass smoke. In addition, there is interest in introducing cleaner fuel types, such as liquid propane gas (LPG), in households that currently use biomass. However, there is little information to date on the effectiveness of these measures in reducing exposures to indoor pollutants. Furthermore, while previous studies have quantified the concentrations of indoor PM and CO resulting from the combustion of solid fuels [21-26], few have attempted to identify biomarkers of such exposures.

Our primary objective was to quantify indoor PM and CO concentrations in an urban city center and its surrounding rural communities. Urban dwellers almost exclusively use LPG for cooking, whereas rural populations primarily use biomass fuels for their domestic energy needs. Furthermore, we sought to evaluate how effective locally made, home-built chimneys are in reducing exposures to indoor pollutants. A secondary objective was to identify chronic vs. acute changes in cardiopulmonary markers associated with exposure to biomass fuel smoke. Specifically, we evaluated exhaled nitric oxide (eNO), exhaled carbon monoxide (eCO), carboxyhemoglobin measured from exhaled breath (eHbCO), oxygen saturation (SpO_2_), carboxyhemoglobin measured from pulse co-oximetry (SpHBCO), and heart rate (HR) to evaluate their potential use as clinical biomarkers for biomass smoke exposure.

## Methods

### Study setting

The study population was comprised of adults ≥18 years of age living in Puno, Peru, and surrounding rural communities, at 3825 meters above sea level. Urban participants were selected from Puno city, commonly work in commerce or education, and use clean fuels, such as LPG, for cooking. Rural participants consist of native subsistence farmers who cook almost exclusively with traditional, open-fire stoves and biomass fuels (i.e., wood, animal dung, and crop residue). The majority of rural kitchens are made of adobe and do not have chimneys. In the vast majority of traditional households, the kitchen is built as a separate building next to the main living area, and the primary cook of the household is almost always female. Cooking is generally performed twice per day, in the early morning and early evening, although the frequency and timing of cooking is somewhat variable. It is not uncommon for women to cook with biomass materials in the morning, then use a gas stove to heat up leftover food for the evening meal. The kitchen windows are small and are usually kept closed, especially in the winter, due to low temperatures at high altitudes. Participants who could read provided written informed consent. Participants who could not read provided verbal consent. The study was approved by the Institutional Review Board of Johns Hopkins University in Baltimore, USA, and A.B. PRISMA in Lima, Peru.

### Study design

We first conducted a door-to-door census of 2,248 urban households in the city of Puno and 1,845 rural households in surrounding communities between July 2010 and September 2010 in specific districts in the department of Puno. We asked questions regarding the location of the kitchen in reference to the main living area, whether the kitchen had a chimney, the type of stove used, and whether or not the household had participated in JUNTOS, a national program providing improved cookstoves to rural residents in Peru [27].

Based on our community census, we selected a random sample of households in three of the following groups (target 30 per group): urban households that predominantly cook with clean fuels (i.e., LPG), rural households that predominantly cook with biomass fuels and have a chimney, and rural households that predominantly cook with biomass fuels but do not have a chimney. Our sample was recruited between January 2011 and November 2011. In total, 104 participants completed a biomass exposure questionnaire. We conducted direct-reading nephelometric PM measurements in 86 homes (27 urban, 28 rural with chimney, 31 rural without chimney). Some rural homes (n = 5) cooked only with LPG during the sampling period; however, these homes used biomass fuels on a regular basis to cook. We chose to include these homes in primary analyses because our main goal was to characterize environmental exposures in rural households of Puno, whether or not they cooked with biomass fuels. None of the urban homes cooked with biomass during the sampling period. We also carried out direct-reading CO measurements in 85 homes, gravimetric PM_2.5_ measurements in 73 homes, and measured cardiopulmonary outcomes in 99 participants of which 76% (n = 75) completed all measurements.

### Measurement of indoor environmental exposures

We measured indoor PM concentrations for a 24-hour period in one-minute intervals in all homes, both urban and rural, using the pDR-1000 in passive mode (Thermo Fisher Scientific, Waltham, MA). Particle monitors were placed 1.5 – 2.0 meters off the ground and 0.5 – 1.0 meters away from the stove. Temperature and humidity were also recorded every minute alongside PM measurements using the HOBO U10 data logger (Onset Corporation, Bourne MA); we adjusted nephelometric PM data for humidity according to previously described methods (28). pDRs were zeroed before each use according to manufacturer instructions.

To quantify coarse and fine fractions of PM, gravimetric measurements of PM_2.5_ and PM_10_ concentrations were obtained concurrently using the DataRAM pDR-1000 converted for active sampling with size-selective inlets and fitted with the PCXR4 universal sampling pump (SKC Inc., Eighty Four, PA) set to a flow rate of 4 L/min and 1.2 L/min for PM_2.5_ and PM_10_ respectively. We also used data from gravimetric samples to develop a calibration curve and convert nephelometric PM values to gravimetric-equivalent PM_2.5_ concentrations. In urban households, gravimetric measurements were conducted for 24-hour periods. In rural households, we conducted gravimetric measurements during morning and evening cook times and when necessary changed the filter using pre-loaded cassettes in order to avoid overloading the filters. Pump flow rates were pre- and post-calibrated in the study office before and after sampling in the field. We excluded filters for which the flow rate change exceeded 25% from all analyses. All filters were weighed pre- and post-sampling in a temperature- and humidity-controlled weighing room at the NIEHS Exposure Assessment Core facility, Johns Hopkins Bloomberg School of Public Health.

We carried out 24-hour direct-reading measurements of indoor CO using a direct-reading instrument in urban and rural homes. We used the EasyLog USB CO Monitor (Lascar Electronics, Erie, PA), which was left in the kitchen during the 24-hour period. Monitors were placed 1.5 – 2.0 meters off the ground and 0.5 – 1.0 meters from the stove in order to approximate the cook’s location.

### Measurement of outdoor environmental exposures

We measured outdoor ambient PM concentrations for a 24-hour period in one-minute intervals twice per month (one weekday and one weekend) from March 2011 through October 2011, using the pDR-1000 in passive mode (Thermo Fisher Scientific, Waltham, MA). Particle monitors were placed on the roof of the study office in the city of Puno. Temperature and humidity were also recorded every minute alongside PM measurements using the HOBO U10 data-logger (Onset Corporation, Bourne MA). We adjusted nephelometric PM data for humidity according to previously described methods (28). pDRs were zeroed before each use according to manufacturer instructions.

### Quantification of household biomass smoke exposure with a questionnaire

We administered a questionnaire to the main cook of all households. We collected information regarding demographics, cooking frequency, time spent cooking on a typical day, stove and fuel type history and typical use, whether participants used an improved stove, and smoking history. We defined biomass burn-years as a measure of cumulative exposure to biomass smoke. One biomass burn-year was equivalent to one hour of biomass use per day multiplied by one year of cooking with biomass, or 365 hours cooking with biomass.

**Table 1:** Census results comparing rural and urban households in Puno

**Census survey**	**Rural (%)**	**Urban (%)**	**p**-**value**
**N** = **4093**	**n** = **1845**	**n** = **2248**	
Is the kitchen connected to the main house?	225(12%)	954 (42%)	<0.001
Does the kitchen have a chimney?	406 (22%)	219 (10%)	<0.001
Do you have an open-fire stove design?	1733 (94%)	362 (16%)	<0.001
Have you participated in the national program for improved cookstoves?	45 (2%)	9 (<1%)	<0.001

### Characterization of household construction

Two or more trained observers assessed aspects of household characteristics including stove type, room volume, the materials of the floor, walls, and roof, the number and area of windows and doors, the state of windows and doors (open or closed), the location of the kitchen and its proximity to living space, and the presence of a chimney.

### Collection of time activity information

In rural households, we visited households during morning and evening cook times. Two or more trained observers were present for the duration of each cook time, and we directly observed the type of stove and fuel being used, the size of windows and doors, and the number of open windows and doors.

### Assessment of cardiopulmonary biomarkers

We visited each household and conducted measurements of eCO, eNO, eHbCO, and SpO_2_ levels on the main cook of the household. The objective of this analysis was to identify chronic vs. acute changes in these clinical biomarkers with exposure to high concentrations of PM and CO produced by the burning of biomass fuels. We chose eNO as a non-invasive measure of airways inflammation. Given that combustion of biomass results in high exposures to CO, we also explored the utility of eCO and SpO_2_ as biomarkers of exposure. Lastly, we chose these biomarkers because they are simple to measure, which is important because of the logistical challenges involved in biomass fuel studies.

We carried out these measurements once in urban households and up to four times in rural households: immediately before and after each of two cooking sessions conducted within 24 hours of each other. eCO and eHbCO levels were collected using the Micro CO Meter (Micro Direct, Lewiston, ME). Measurements of eNO were taken using the NIOXMINO Airway Inflammation Monitor (Aerocrine, Solna, Sweden), and levels of SpO_2_ and SpCO in the blood were assessed using the Rad57c carboxyoximeter (Masimo Corp, Irvine, CA).

### Biostatistical methods

We used linear regression methods to generate a calibration equation using gravimetric and humidity-corrected passive nephelometric measurements [28] representing 24-hour averages in urban households and cook time averages in rural households. We log-transformed both gravimetric and nephelometric measurements in generating the equation.

We conducted basic comparisons of census data and demographic and household characteristics using Fisher’s exact test and the Kruskal-Wallis test, where appropriate. We compared environmental exposures (24-hour average PM, 24-hour CO, cook time PM, cook time CO) and physiological outcomes (eNO, eCO, eHbCO, SpCO, SpO_2_) using the Mann Whitney U test, and we compared physiological outcomes before and after cooking using the Wilcoxon Signed-Rank test. Approximately 30% of eNO levels after cooking were missing because of difficulties using the machine in cold temperatures. In our comparison of outcomes in urban and rural participants, we used the averages of all outcome measurements conducted on rural participants (one measurement before and one after cooking for each of two cook sessions, for a total of four measurements). In our comparison of outcomes before and after cooking in rural participants, we used the average of measurements before cooking (up to two per participant) and average of measurements after cooking (also up to two per participant). Measurements were conducted only once on urban participants.

We used multivariable linear regression to evaluate the association between PM concentrations (24-hour average and cook time) and kitchen and cooking factors in rural houses only. This analysis excluded urban homes and rural homes that used LPG for cooking. We used generalized estimating equations to account for correlation between morning and afternoon measurements within each household. The final model was selected using a quasi-likelihood information criterion (QIC) approach. We log-transformed both 24-hour average and cook time PM concentrations because they were right skewed. We excluded rural households that cooked with gas in this analysis because they were few in number and because we were most interested in how household factors relate to PM generated from biomass fuel smoke.

We conducted our analyses in R (http://www.r-project.org) and STATA 11 (Stata Corp., College Station, Texas).

## Results

### Characteristics of the study population

Responses to the household census regarding kitchen and stove characteristics are summarized in Table 1. A small proportion of rural households reported having a chimney, and an even smaller percentage of respondents had participated in the national improved cookstove program that provides a cash incentive for households who build an improved cookstove [27]. In Table 2, we summarize demographic and household characteristics in participating urban and rural households. Nearly all main cooks across groups were female, and cooks in rural homes with chimneys were slightly younger than other groups. A large majority of rural cooks used a traditional cookstove at every meal, whereas no urban cooks did so. However, some urban participants (9.4%) used traditional stoves on occasion. Lifetime cumulative exposure to biomass was significantly higher in rural participants as compared to urban participants. A small proportion of households had improved cookstoves in our study population. Only one participant in our study smoked on a daily basis, and overall lifetime cumulative exposure to smoking was negligible.

**Table 2:** **Demographic and household characteristics in study participants living in urban households**, **and rural households with and without chimneys**

	**Mean (SD) or %**				
**Characteristic**	**Total**	**Urban**	**Rural-chimney**	**Rural-no chimney**	**P-value**
	**(n=104)**	**(n=32)**	**(n=34)**	**(n=38)**	
**Demographics**					
Age (years)	48	51	43	52	0.03
Sex (% female)	98.1	100	100	94.7	0.33
**Cooking history**					
Years cooking	32	31	29	36	0.17
Years cooking with traditional stove	24	5	28	35	< 0.001
Biomass burn-years^*^	116	16	84	226	< 0.001
**Stove and fuel use**					
Hours spent cooking per day	3.9	3.0	2.9	5.5	0.17
Cooking frequency, % who cook every day	96.1%	90.6%	97.1%	100%	0.07
Cooking frequency per day, n (%)					< 0.001
1	9 (8.7)	6 (18.8)	1 (2.8)	2 (5.6)	
2	69 (66.4)	7 (21.9)	31 (86.1)	31 (86.1)	
3	26 (25.0)	19 (59.4)	3 (8.8)	4 (10.5)	
% cooking every meal with a traditional cookstove	55.8	0	79.4	81.6	< 0.001
% using a traditional cookstove (any frequency)	70.2	9.4	100	94.7	< 0.001
Biomass fuel sources (participants could select ≥1), n (%)					
Wood	72 (69.2)	8 (25.0)	31 (91.2)	33 (86.8)	< 0.001
Dung	75 (72.1)	8 (25.0)	31 (91.2)	36 (94.7)	< 0.001
Agricultural materials	36 (35.0)	0 (0)	15 (44.1)	21 (56.8)	< 0.001
**Improved cookstoves**					
Has improved cookstoves, n (%)	5 (4.8)	1 (3.1)	3 (8.3)	1 (2.8)	0.62
Uses improved stove regularly	1	0	1	0	
Improved cookstove via JUNTOS, n (%)	0 (0)				
**Smoking history**					
Current daily smokers, n (%)	1 (0.9%)	1 (2.9%)	0	0	
Cumulative pack-years among all participants	0.08	0.22	0	0.02	0.34
**Kitchen characteristics**					
% with kitchen separate from main living area	78.6	29.0	100	100	< 0.001
Volume of kitchen (cubic meters)	24.0	30.5	22.8	19.6	0.06
% with a dirt floor	66.0	12.9	85.3	92.1	< 0.001
% with adobe walls	66.0	9.7	97.1	84.2	< 0.001
Roof material, n (%)					< 0.001
Concrete	22 (21.4)	21 (67.7)	0 (0.0)	1 (2.6)	
Corrugated iron	44 (42.7)	7 (22.6)	22 (64.7)	15 (39.5)	
Thatch	29 (28.2)	0 (0.0)	11 (32.4)	18 (47.4)	
Other	8 (7.8)	3 (9.7)	1 (2.9)	4 (10.5)	

### Calibration equation of nephelometric to gravimetric PM_2.5_ concentrations

**Figure 1:**
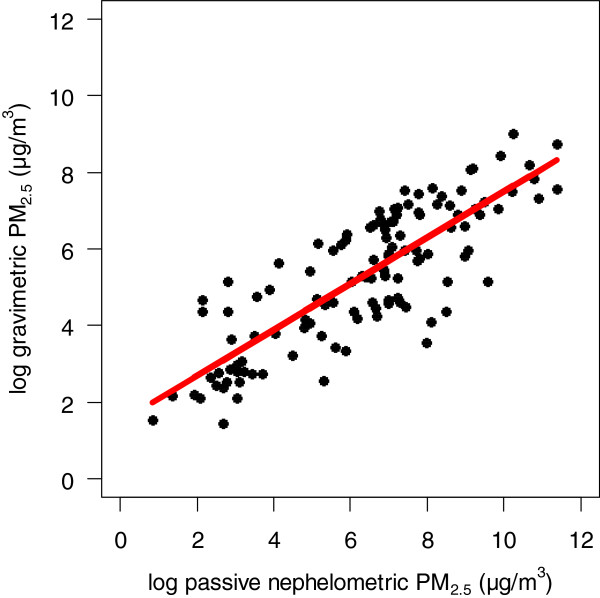
**Plot of log**-**transformed gravimetric PM**_**2.5 **_**concentrations vs. log**-**transformed passive nephelometric PM concentrations**. The red line represents the calibration equation: Gravimetric PM_2.5_ = *e* ^1.5 + 0.6 × ln(nephelometric PM)^.

Gravimetric PM_2.5_ and nephelometric PM concentrations were linearly related in the log scale (Figure 1). Although nephelometric PM concentrations overestimated gravimetric PM_2.5_ concentrations on average by 50%, the relationship was consistent and could be predicted with the following equation: Gravimetric PM_2.5_ = *e* ^1.5 + 0.6 × ln(nephelometric PM)^ This equation was used to convert nephelometric PM concentrations to PM_2.5_ equivalent concentrations.

### Household environmental exposures

**Figure 2:**
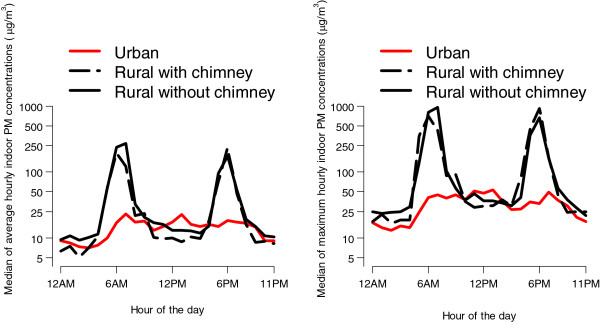
**Median (left) and maximum (right) average concentrations of indoor PM by hour of the day.** Curves are stratified by site and chimney construction (urban, rural with chimney, and rural without chimney). The y-axis represents summaries of average hourly concentrations (in μg/m^3^) of indoor PM across households. We calculated these household summaries for each hour of the day, as indicated in the x-axis.

Median, average, and maximum hourly PM concentrations were substantially lower in urban houses vs. rural houses throughout the course of a 24-hour day (Figure 2). In rural homes with and without chimneys, PM concentrations peaked at approximately six in the morning and six in the evening, corresponding with observed practices regarding cook times during the day. We observed smaller, less distinct maximum concentrations in urban households, although concentrations tended to be higher overall in the daytime hours and peaked slightly at seven in the morning, one in the afternoon, and seven in the evening. We found little difference in exposure between rural homes with and without chimneys (p=0.40).

**Figure 3:**
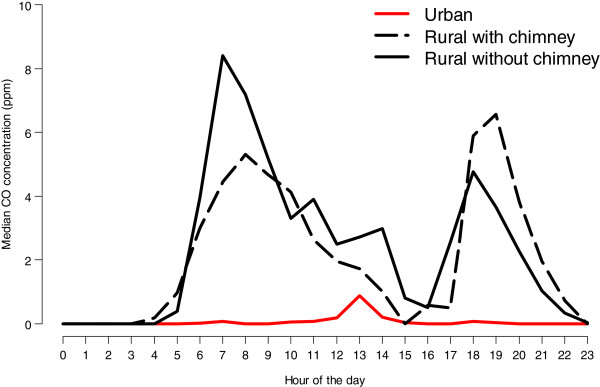
**Median indoor CO concentrations by hour of the day.** Curves are stratified by site and chimney construction (urban, rural with chimney, and rural without chimney). The y-axis represents median values of indoor CO (ppm) across households. We calculated these household summaries for each hour of the day, as indicated on the x-axis.

Median average hourly CO concentrations were also substantially lower in urban houses vs. rural houses (Figure 3). Maximum CO concentrations in rural households and trends mimic those of PM concentrations; however, CO concentrations decreased from their maxima more slowly than PM concentrations over time (i.e., CO concentrations remained persistently higher for a longer period than did PM concentrations before returning to zero). There is a slight increase in urban household CO concentrations at approximately one in the afternoon.

Median 24-hour average PM_2.5_ equivalent concentrations were 22 vs. 130 μg/m^3^ (p<0.001) and CO concentrations were 0.4 ppm vs. 5.8 ppm for urban vs. rural houses (p<0.001), respectively. Median 24-hour PM and CO concentrations were 119 μg/m^3^ and 137 μg/m^3^ (p=0.40) and 4.6 ppm and 7.2 ppm (p=0.30) for rural houses with and without chimneys, respectively. Median cook time PM and CO concentrations were 298 μg/m^3^ vs. 360 μg/m^3^ (p=0.45), and 9.4 ppm vs. 15.2 ppm (p=0.23) in rural houses with and without chimneys, respectively. The Pearson correlation coefficient between 24-hour average PM and CO concentrations for urban households was 0.07 (95% CI 0.33 to 0.44), and for rural households was 0.67 (95% CI 0.49 to 0.79). Pearson correlation coefficients for rural houses with chimneys and rural houses without chimneys were 0.54 (95% CI 0.21 to 0.76) and 0.79 (95% CI 0.52 to 0.92), respectively.

Median gravimetric 24-hour PM_10_ and PM_2.5_ concentrations were 30 μg/m^3^ and 13 μg/m^3^ for urban households, respectively, for a coarse fraction of 17 μg/m^3^. Median gravimetric cook time PM_10_ and PM_2.5_ concentrations in rural houses were 561 μg/m^3^ and 316 μg/m^3^, respectively, for a coarse fraction of 245 μg/m^3^. These data suggest that fine PM represents 43% (urban) to 56% (rural) of the exposure at our study sites.

### Outdoor environmental exposures

Monthly 24-hour average PM_2.5_-equivalent concentrations ranged from 18 μg/m^3^ (March) to 29 μg/m^3^ (June), with an overall median of 23 μg/m^3^ during the study period.

### Factors associated with higher environmental exposures

**Figure 4:**
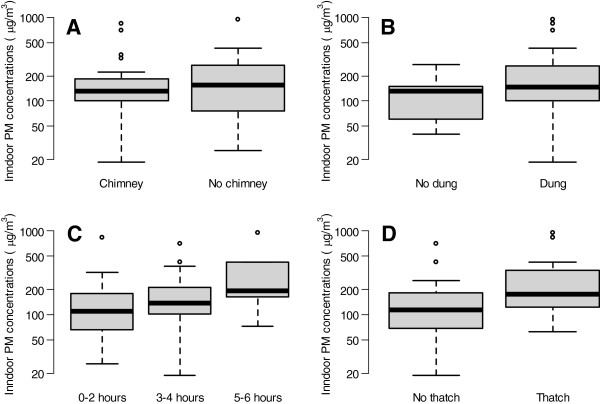
**Boxplots of 24**-**hour indoor PM concentrations by household characteristics among rural participants.** Presence of a chimney **(panel A)**, use of dung while cooking **(panel B)**, number of hours cooking **(panel C)**, having a thatch roof **(panel D)**.

We identified four factors that were potentially associated with higher 24-hour average PM concentrations in multivariable analyses and after model selection using the QIC (Figure 4). Having a thatched roof (p=0.007) and the number of hours cooking on a typical day (range: 1 to 6 hours per day, p=0.02) were positively associated with increased 24-hour PM concentrations; however, neither having a chimney (p=0.87) nor using dung for cooking (p=0.17) were significantly associated with higher 24-hour average PM concentrations.

### Cardiopulmonary biomarkers associated with biomass fuel exposure

**Figure 5:**
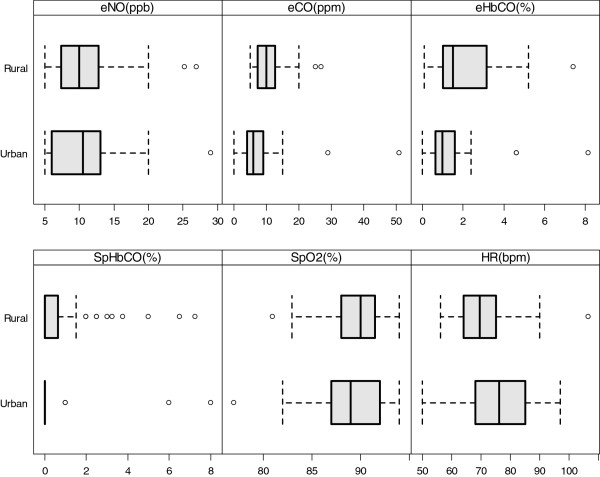
**Boxplots of cardiopulmonary outcomes stratified by site (rural vs. urban).** Displayed are exhaled nitric oxide (eNO), exhaled CO (eCO), carboxyhemoglobin from exhaled breath (eHBCO), carboxyhemoglobin from pulse co-oximetry (SpHbCO), pulse oximetry (SpO_2_) and heart rate stratified by site (rural vs. urban).

We display results of several outcome measurements in urban vs. rural participants in Figure 5. In urban vs. rural participants: median eNO levels were 10.5 ppb vs. 10.0 ppb (p=0.64); median eCO levels were 6 ppm vs. 9.5 ppm (p=0.01); and, eHbCO levels were 0.96% vs. 1.56% (p=0.04); median SpHbCO levels were 0% vs. 0% (p=0.13); median SpO_2_ levels were 89% vs. 90% (p=0.41); and, median heart rates were 76.0 vs. 71.3 beats/minute (p=0.06), respectively. The Pearson correlation coefficient for eCO versus 24-hour average kitchen CO concentrations was 0.29 (95% CI 0.07 to 0.48). In Table 3, we show median values for several biomarkers in rural participants before and after cooking. eNO increased by 2 ppb (p=0.006) and SpO_2_ decreased by 1% (p=0.02) significantly after cooking; however, the magnitudes of these changes were small.

**Table 3:** Median values of biomarkers in rural participants before and after cooking

	**Before cooking**	**After cooking**	**P-value**
eNO (ppb)	9	11	0.006
eCO (ppm)	9	10	0.54
HbCO (%)	1.4	1.6	0.22
SpCO (%)	0	0	0.25
SpO_2_ (%)	90.3	89.3	0.02
Heart rate	69.5	71.8	0.12

## Discussion

Participants living in rural homes cooking primarily with biomass fuels experienced daily indoor PM concentrations that were 6-fold higher than participants living in the urban households in Puno. These PM exposures were up to 5.5-fold higher than the 24-hour World Health Organization (WHO) safe air quality standard of 25 μg/m^3^[29]. Measurements of ambient PM concentrations show that background ambient PM concentrations are not likely to contribute heavily to the exposures experienced by those cooking with biomass. There has been a growing interest in the health effects of the coarse fraction, defined as the fraction of particles between 2.5 μm and 10 μm in diameter [30,31]. Results of gravimetric analyses demonstrate that biomass fuel smoke had a slightly higher proportion of fine PM. In contrast, PM in urban households contain a slightly higher proportion of coarse PM, most likely contributed by ambient PM from outdoor sources. Our results are comparable to concentrations found in other studies conducted in regions where biomass fuel use is highly prevalent [32]. On average, rural participants in our study reported cooking with a traditional cookstove for 32 years, indicating that women in this region experience extremely high lifetime cumulative exposures to biomass smoke. Furthermore, since children are often present inside the kitchen during cooking, lifetime exposure may be even higher than our estimates, especially for women. These high cumulative exposures to biomass fuel smoke signify that individuals in this region are at elevated risk of many communicable and non-communicable chronic diseases associated with exposure to environmental pollutants found in biomass smoke.

While PM and CO concentrations were somewhat elevated in homes without chimneys as compared to rural homes with chimneys, we did not see a significant difference in these concentrations, indicating that locally constructed chimneys were minimally effective at reducing exposures to biomass smoke in our population. Chimney design and installation varied considerably across households. Specifically, we observed chimneys that were both vertically and horizontally oriented, constructed of a variety of materials including polyvinyl chloride pipe, adobe, and brick. Some stoves were placed in a recessed area of the kitchen wall while others were not. While observing differences in exposures resulting from different forms of chimney construction is beyond the scope of this study, it is important to highlight the importance of instruction and supervision for proper construction of chimneys at the local level for future policy and implementation studies. There is a strong need to incorporate technical assistance for appropriate construction of chimney stoves in cookstove intervention programs and to evaluate the effectiveness of chimney design.

Data from our census demonstrate that at the time of our study, government-sponsored cookstove intervention programs had yet to reach many communities in the region of Puno, with only 2.4% of households reporting participation in the national improved cookstove program through JUNTOS [27]. Similarly, only five participants in our sample reported having an improved stove, and none reported having received this stove from the JUNTOS program. Only 22% of households in our census reported having chimneys in their kitchens. While it is still unclear exactly what an effective improved cookstove intervention should consist of, attention should be given to this region of Peru where there is a dire need for effective cookstove intervention programs.

We found that eNO and SpO_2_ changed significantly in rural participants after cooking as compared to before cooking; however, the magnitudes of these changes were negligible and not biologically meaningful. Although eCO levels were weakly but significantly correlated with 24-hour CO concentrations, we found a significant difference in eCO levels between urban and rural participants. This reflects the differences in indoor CO concentrations found in urban and rural kitchens. These differences remained after eliminating the only daily smoker from the analysis. The low prevalence of current and lifetime daily smoking suggests that differences in these levels can be attributed to differences in exposure to biomass fuel smoke. While urban participants were slightly older than rural participants, we do not believe that this affected the differences in eCO levels. Other studies have also demonstrated the utility of eCO as a biomarker for biomass smoke [33,34]. However, its utility may be limited somewhat by the relatively short half-life of CO in the body, approximately two to 4.5 hours, depending on initial exposure [35]. In our study, since we measured eCO immediately before and after cooking in rural households, we were able to capture eCO levels within this critical window. We saw a significant difference in eCO levels between urban and rural participants, but did not observe a significant difference before as compared to after cooking. These observations suggest that eCO could represent a useful personal biomarker for long-term, chronic exposure to biomass fuel smoke. An exploration of concentrations of CO generated under different combustion conditions is an important validation step that would be needed to employ eCO as a marker for biomass combustion. However, in our study, biomass composition and combustion conditions were similar in all rural homes and the use of LPG was predominant in our urban homes. Therefore a discussion of the potential contributions of CO generated by different sources or combustion processes were beyond the scope of this analysis. eCO is simple and inexpensive to measure, requiring the participant to simply hold his or her breath for 20 seconds and then exhale fully into the machine. This simplicity is particularly useful because the instrument can be taken to the homes of the participants, instead of requiring the participant to travel to a local clinic for measurement. Hence, eCO may represent a useful and practical biomarker for biomass fuel smoke exposure and warrants further exploration for use in cookstove intervention studies.

Participants in rural areas who had a chimney installed in their homes tended to be younger than those living in homes without a chimney by about 9 years. Furthermore, rural participants who occasionally cooked LPG were younger by 9 years. While these differences may simply be a result of chance given the relatively small sample size, they also may reflect a trend for younger families to adopt these technologies. Younger families may also represent a willing target population for intervention.

Not surprisingly, we found that kitchen PM concentrations increased with the number of hours participants reported cooking on a typical day. In addition, although not statistically significant, we found that using dung for fuel also increased PM concentrations. We were unable to separate the effects of different types of biomass fuels (e.g. wood, dung, crop waste) on PM concentrations because most participants used multiple fuel sources for cooking. Since we excluded LPG households from the analysis, the increase in PM levels compares households that used dung with households that used other forms of biomass. Thus, our results suggest that dung use leads to a modest increase in exposure to PM even as compared to other biomass fuels. Contrary to our expectations, we found that kitchens with thatched roofs had slightly higher levels of indoor PM than other roof materials such as corrugated metal. These results could be due to the fact that thatched roofs were often tightly constructed and may have resulted in less ventilation than metal roofs. Furthermore, thatched roofs can hold a considerable amount of previously deposited PM, which can come loose when agitated or brushed by people working inside the kitchen. There has been considerable interest in the potential for using CO as a proxy for PM_2.5_ in biomass fuel studies [36]. Our results support this relationship in that kitchen CO concentrations were positively and significantly correlated with PM_2.5_ concentrations in rural households.

An important strength of our study is that two or more trained observers were present during the cooking period in rural homes, allowing us to directly record household characteristics and cooking behaviors rather than relying on self-report. There are also limitations to our study. First, concentrations measured in a household kitchen may not represent personal exposures. In particular, we may be underestimating personal exposures to environmental pollutants in urban participants given that they are likely exposed to higher concentrations of ambient PM when outside the home. Outdoor PM concentrations in rural areas are quite low, and thus kitchen exposures are likely to be the predominant source of PM exposure for rural participants. However, time spent indoors is a significant modifier of personal exposure. Second, we only recorded kitchen concentrations of household air pollution for a 24-hour period. Given the high intra-household variation found in other studies [37], longer monitoring periods would have provided more data to evaluate this variability. Third, we were unable to stay for the full duration of fuel combustion, and in most instances we were unable to determine whether participants remained in the kitchen until the fire was completely extinguished.

It is possible that participants changed their cooking behaviors as a result of our presence in their homes. Regardless, we believe that this effect was not a great source of error and we prefaced all of our visits by emphasizing that we wanted them to cook as they normally do.

Another important strength of our study is that we conducted gravimetric PM_2.5_ measurements concurrent with passive, real-time nephelometric measurements in order to validate and generate a calibration equation for passive measurements against a gold standard. Real-time optical devices, such as the pDR-1000 used in this study, are thought to overestimate PM_2.5_ mass compared to gravimetric assessments [38]. We found in our study that the pDR-1000 tended to underestimate PM at very low concentrations as compared to gravimetric measurements and overestimated PM at higher concentrations, following a logarithmic function. There are important limitations to the validation component of our study. First, due to logistical limitations, we pre- and post-calibrated pumps in the study office, as opposed to calibrating immediately before and after deployment in the field, which may have led to variable accuracy in recorded pump flow. Second, we observed breakthrough PM on many of the filters, meaning that our calibration equation most likely yields conservative concentration estimates after correction. However, our study is valuable and novel in that it investigates the use of a real-time optical instrument (the pDR-1000) that is simple to use in a setting where it is logistically challenging to carry out the gold standard (gravimetric) measurement method.

## Conclusions

Environmental exposures due to biomass fuel smoke were several-fold greater in rural vs. urban households, and having a chimney did not significantly reduce these exposures in rural households. Technical assistance for appropriate stove and chimney construction should be a key component of intervention programs. Measurement of eCO may represent a useful and easily measurable cardiopulmonary biomarker for chronic exposure to biomass fuel smoke in future studies.

## Abbreviations

PM: Particulate matter; CO: Carbon monoxide; COPD: Chronic obstructive pulmonary disease; LPG: Liquid propane gas; eNO: Exhaled nitric oxide; eCO: Exhaled carbon monoxide; eHbCO: Carboxyhemoglobin measured from eCO; SpO2: Oxygen saturation; SpHBCO: Carboxyhemoglobin measured from pulse co-oximetry; HR: Heart rate; QIC: Quasilikelihood information criterion.

## Competing interests

The authors have no competing interests to declare.

## Authors’ contributions

SLP contributed to study design, coordinated data collection, conducted statistical analyses, and drafted the manuscript. DLW and PNB contributed to study design and drafting of the manuscript. PAB assisted in study design and data collection. LMG assisted in data collection. RHG and JJM contributed to drafting the manuscript. WC conceived of the study and contributed to study design, statistical analyses, drafting of the manuscript and had ultimate oversight over study design and administration. All authors approved the final manuscript.

## Other members of the CRONICAS Cohort Study group

Antonio Bernabé-Ortiz, Juan P. Casas, George Davey Smith, Gregory B. Diette, Shah Ebrahim, Raúl Gamboa, Héctor H. García, Luis Huicho, Fabiola León-Velarde, Germán Málaga, Víctor M. Montori, María Rivera Ch., Katherine Sacksteder, Liam Smeeth; Robert A. Wise.
